# Shaping illiberal citizenries: Far-right justifications of educational structures

**DOI:** 10.1177/14749041241308600

**Published:** 2024-12-24

**Authors:** Anja Giudici, Anna Pultar

**Affiliations:** Cardiff University, UK; Heriot Watt University, UK

**Keywords:** Far right, tracking, comparative education, politics of education, equality, populism

## Abstract

Educational structures do not only have pedagogical implications; by defining and ranking categories of teaching and learning, and by assigning individuals to these categories, they also contribute to shaping the identity and stratification of citizenries. It is this latter dimension that is often mobilised in the political debate on structures. Existing literature in sociology and politics shows that the main parties in post-World War II Europe have typically invoked liberal citizenship norms based on solidarity, autonomy and pluralism in this debate to appeal to both domestic electorates and international audiences. But what about those who disagree with liberal and egalitarian understandings of citizenship? This paper examines one such movement: the post-1945 Western European far right. Applying rigorous content analysis to an extensive original database of archival documents, we show that extreme and radical right parties and intellectual organisations largely advocate highly stratified education systems and justify this preference with social-order based citizenship norms – but we also find rhetorical variation. While existing theories of liberal-democratic education politics can serve to understand some of this variation, more specific theorising may be needed for educational research to develop a finer-grained understanding of the rhetorical and positional choices of actors who reject liberal-democratic principles.

In 1946, US President Harry Truman appointed a commission to investigate how education might have contributed to the rise of National-Socialism in Germany. The commission pointed the finger not only at the content but also at the structure of German education, particularly its hierarchical stratification. Educational structures, it argued, shape citizens’ participation and identification with society. In the case of Germany, early streaming and unequal quality standards had developed ‘an attitude of superiority among a small elite and a sense of inferiority among the majority of Germans’ (quoted in Edelstein, 2020). For the country to develop into a democracy, they had to go.

In the 1940s, highly stratified and non-standardised educational structures were not unique to Germany. But they were soon to change. With varying degrees of intensity, post-1945 reforms reshaped the structure of Western European education systems in two common directions. A first set of policies targeted stratification. They mitigated steep tracking and gradually moved towards making pupils’ talent, rather than their socio-economic background, gender and ethnicity, the formal criterion for academic selection (Furuta, 2020; Giudici et al., 2023; Österman, 2018; Wiborg, 2009). A second type of reform increased the standardisation of schooling, with new regulations on content, enrolment and standards homogenising provision across schools and regions (Allmendinger, 1989; Gingrich, 2011).

Scholars identify various drivers behind stratification and standardisation reforms, including socio-economic rationales (Sass, 2022), global norms (Furuta, 2020), and electoral concerns (Gingrich, 2011; Giudici et al., 2023; Wiborg, 2009). There are also lively debates about the pedagogical implications of tracking (Rubin and Noguera, 2004). However, reformers themselves – like the Truman Commission before them – have often rhetorically linked their preferences and actions to their vision of citizenship when justifying their preferences on structures. The centre-left presents comprehensive education and equalised standards as a means of enabling individuals to autonomously participate in a more united and solidary citizenry (Esping-Andersen, 1990; Wiborg, 2009). Instead, centre-right parties have called for improved standards, opportunities and bridges across tracks within a structurally diversified system. They justify such preferences with the need to strengthen social pluralism and participatory autonomy (Giudici et al., 2023; Österman, 2018).

The two positions are different. But they both relate to citizenship norms – that is, ideas about how citizens should understand and participate in society. More specifically, these arguments refer to citizenship norms that political theorists associate with ideologies that adhere to liberal democratic principles (Dalton, 2008; Heater, 1990; Wallace Goodman, 2022). They suggest that educational structures should encourage more autonomous (centre-right) or solidary (centre-left) forms of participation. And they advocate a mix of unitary and pluralist understandings of society.

A combination of two literatures can help us theorising these argumentative differences and similarities. Work in the sociology of education shows that liberal-democratic citizenship norms became globally dominant in the post-World War II period, and have influenced educational reforms ever since (Furuta, 2020; Lerch et al., 2022). Political actors therefore often refer to such dominant norms, either because global norms have actually shaped their thinking or because they seek legitimacy from domestic and international audiences who accept them. At the same time, political scientists suggest that parties participating in electoral contests align both preferences and rhetoric with those preferred by the specific constituencies they represent (Ansell, 2010; Gingrich, 2011). More stratified and de-standardised education systems, as well as citizenship norms that emphasise pluralism and participatory autonomy, tend to appeal to the more socially advantaged constituencies of the centre-right, while the less well-off may be more attracted by promises of equal structures and solidarity.

These literatures assume that political actors are committed to liberal norms and democratic politics. While this assumption applies to the traditionally dominant forces of post-1945 Western European politics on which research has focused, it does not hold for all. A particularly powerful opponent of liberal democracy in contemporary Europe is the far right, whose ideology is based on authoritarianism, nativism, and either illiberalism or anti-democracy (Carter, 2018; Mudde, 2019). Such ideological tenets are typically associated with a unitary social order-based understanding of citizenship that emphasises individuals’ duty to participate according to their assigned roles and to identify with a unitary, anti-pluralist understanding of society (Dalton, 2008; Denters et al., 2007).

Illiberal citizenship norms have been largely discredited in post-WWII politics (Bar-On 2007). Do representatives of the post-1945 Western European far right still rhetorically relate their preferences for educational structures to such norms? If so, can the quantitative patterns and the qualitative nature that characterise this relationship be understood through the lens of existing sociological and political theories of education politics?

The study answers these questions in three steps. First, we combine sociological and political work on education to develop an analytical framework theorising how centre-right and centre-left parties rhetorically link educational structures and citizenship norms (Section 1). By linking this framework to studies of the far right, we develop expectations about how this rhetorical link may vary across the, internally diverse, far-right movement (Section 2). We then confront these expectations with a novel database of far-right sources, covering all the major parties and the most influential intellectual organisations of the post-WWII Western European radical and extreme right. To document the views of the former, we rely on political manifestos, while to analyse the views of intellectuals, we use publications from official far-right publishing houses. Section 3 outlines our methodology.

The analysis in Section 4 shows, first, that the contemporary far-right is not a single-issue movement (Mudde, 2019). It confirms findings from the emerging literature on far-right approaches to education, which show that the movement has developed specific education-related positions and strategies. While existing research tends to emphasise the movement’s interest in the shaping the curriculum (Berg et al., 2023; Neumann, 2022), this paper finds that it also has consistent, but varying, preferences on educational structures. We further show that far-right actors invoke citizenship norms to justify these preferences, and that factions within the movement that are more sensitive to the electoral and global logics theorised in the existing literature, are less likely to rhetorically invoke illiberal citizenship norms.

Understanding how the far right approaches education is important both empirically and theoretically. As educational research on the far right is still in its infancy (Berg et al., 2023; Giudici, 2021), we know little about the educational preferences and strategies of a movement that studies have shown is capable of shaping European politics both directly, by taking power, and indirectly, by redefining the norms and discourses to which political actors refer (Abou-Chadi and Krause, 2020; Valentim, 2024). Moreover, studying the far right – and other opponents of liberal-democracy – can serve to refine and test the limits of existing theories, many of which are explicitly or implicitly based on insights from the traditionally dominant parties and progressive movements (Niesz et al., 2018).

Both arguments are particularly relevant to educational structures. Reforms of tracking, selection, and equalisation of standards and prestige target highly visible aspects of education with deep redistributive and cultural implications. They have repeatedly proved to be a particularly controversial and effective means of bringing people onto the streets and swinging elections (Ambler, 1994; Giudici et al., 2023). They are therefore of strategic importance to political movements. A wealth of empirical research also demonstrates that the link between the structure of education and of society is not just rhetorical. Structural reforms have significant implications for social outcomes, ranging from economic and political inequality (Brunello and Checchi, 2007; Werfhorst and van de Mijs, 2010) to students’ self-worth and identity (Khan 2011) and political views (Chin, 2025; Gingrich, 2019; Mendelberg et al., 2017). Understanding how parties approach and justify structures is important and provides an insight into both their educational and societal views.

## Educational structures and citizenship norms in political rhetoric

Structures are an inherent feature of education systems. In contrast to informal learning arrangements, formal education is defined by organisational differentiation, that is, by institutionalised means of determining the distribution of educational content, credentials and opportunities across the student population (Allmendinger, 1989; Sørensen, 1970). To conceptualise structures, we draw on Allmendinger (1989), who first theorised stratification and standardisation as two constituent sub-dimensions of educational differentiation. These concepts have proved particularly useful in studies comparing educational structures and related debates across space and time.

Stratification describes the degree of tracking (Allmendinger, 1989; Sørensen, 1970). Highly stratified education systems separate students early into regulated streams that differ in terms of curricula, teaching methods, and certificates. They also use selective procedures that rank tracks and ensure that only a limited proportion of pupils can reach the top of the academic ladder, either on the basis of academic talent or criteria such as gender, class, or race. In contrast, less stratified systems are characterised by less tracking and selection.

Standardisation refers to the degree of homogeneity of educational provision (Allmendinger, 1989; Jackson, 2013). In highly standardised systems, state authorities ensure that students across the territory receive the same educational content and quality through interventions in curricula, funding, teacher training and examinations. As a result, certificates give more homogeneous signals to employers, education providers, and society. Less standardised systems leave such decisions either to local authorities or, in the case of functional devolution, to schools (Gingrich, 2011), resulting in more heterogeneous schooling.

Although they describe different ways of structuring education, standardisation and stratification can act as substitutes. For example, research on the US shows that after court decisions in the 1950s outlawed racial stratification, some states de-standardised their education systems by deregulating private education and student admissions. De facto, these policies served to perpetuate features of racial stratification by allowing (white and religious) parents to choose the content and peers with whom they wanted their children to interact, and by perpetuating funding disparities (Hackett, 2020; Rooks, 2017).

Educational structures serve primarily practical and pedagogical purposes. They divide pupils into streams, types and levels, thus making the student population bureaucratically and pedagogically manageable. They also structure pedagogical practices and the teaching profession (Mayer et al., 2018). However, sociologists argue that structures also have a social and political dimension. They define and rank categories of education, which translate into ‘powerful social categories’ (Domina et al., 2017: 311; see also Brighouse, 2006). For example, someone assigned to a less academically demanding track will have different expectations of their social, economic and political role than someone with a prestigious university degree. Educational structures therefore both organise citizenship and legitimise its organisation. Or, as put by Marshall (1965: 110), ‘the status acquired by education is carried out into the world bearing the stamp of legitimacy, because it has been conferred by an institution designed to give the citizen his [sic] just rights’.

Work in the politics of education finds that it is this citizenship-related dimension that parties mobilise rhetorically when they engage in debates about structures (Busemeyer, 2014; Gingrich, 2011; Giudici et al., 2023, Wiborg, 2009). This work also shows that structures are among the most contested issues in post-1945 European education politics, with both expressed preferences and their justifications varying systematically across the dominant party families.

More specifically, left-wing parties have typically supported state interventions aimed at standardising (Gingrich, 2011) and de-stratifying education (Österman, 2018; Pultar, 2021; Wiborg, 2009). Their main argument for this preference is that more inclusive structures and equal standards increase social mixing and enable the less fortunate to exercise their citizenship rights, thus fostering a more unified and solidary citizenry (Pultar, 2021). As the Austrian Social Democrats stated in 2008: ‘Full participation first requires integrated forms of education across all school levels’ (SPÖ, 2008). Left-wing support for (specific forms of) de-standardisation – such as school autonomy – since the 1990s has been justified on similar grounds, with the need to equalise community participation in a pluralist society (Gingrich, 2011).

Especially conservative parties historically opposed de-stratification and standardisation (Ansell and Lindvall, 2021). After the Second World War, however, they partially abandoned this position. Since then, centre-right parties have instead typically advocated expanding opportunities and bridges across tracks, while working to protect softer forms of stratification as well as the autonomy of individuals, schools, and private providers from state-prescribed standards. Their central justification is that education should be structured to equalise opportunities, but also to encourage people to seize such opportunities autonomously and to develop plural forms of identity within a unified society. As a representative of the Italian Christian Democrats argued in a parliamentary debate, structures should level the playing field but also allow society to ‘discriminate between those with genius and those without’ (Limoni, 1962: 36265).

This debate mobilises the citizenship norms that political theorists associate with liberalism, or the notion that individuals are ‘the sole intrinsic objects of moral concern’ and should enjoy equal rights (Brighouse, 2006: 5). Political theorists define citizenship norms as beliefs about what just rights citizenship requires and how good citizens should use them (Dalton, 2008). Such beliefs are found to vary along two dimensions: first, how citizens should participate in society and, second, how they should identify with it (Dalton, 2008; Heater, 1990; Wallace Goodman, 2022).

With regard to participation, this literature typically distinguishes three alternative norms: solidarity, autonomy, and social order (Almond and Verba, 1963; Dalton, 2008; Denters et al., 2007). The first two draw on different interpretations of liberalism. Solidarity sees inequalities of status or resources as limiting the right and ability of individuals to participate, and thus emphasises mutual support as a core component of being a good citizen (Heater, 1990; Marshall, 1965). We can see this norm in left-wing arguments for structures that promote equality and social mixing as pathways to inclusive participation.

A second interpretation of liberalism does not problematise inequality as long as individuals are given opportunities to participate. It therefore emphasises individuals’ autonomy in seizing opportunities as core component of good citizenship (Dalton, 2008; Denters et al., 2007). This norm underpins centre-right parties’ justification of structures that equalise opportunities and nurture talent (Gingrich and Giudici, 2023).

Missing from this debate is the third understanding of participation, the social order norm (sometimes referred to as duty-based or traditional elitist participation norm). It emphasises the duty of individuals to contribute according to their role in an ordered society and is typical of illiberal ideologies that reject individual rights and equality (Almond and Verba, 1963; Dalton, 2008; Denters et al., 2007).

The second dimension of citizenship norms concerns beliefs about how individuals should identify with society. In her work, Wallace Goodman (2022) distinguishes two alternative identification norms. Unitarism holds that everyone must identify with a community in the same way, while pluralism accepts that different forms of identification can and should form the basis of citizenship. The positions above show a mixture of both, with left-wing parties typically emphasising unitarism and centre-right parties foregrounding pluralism. Neither camp rhetorically relies solely on unitarism to the exclusion of individual rights.

How can these patterns be theorised? Two dominant approaches have emerged in the education literature, both of which draw on insights derived from the dominant forces in post-1945 Western politics. We argue that, together, they provide a valuable framework for theorising how political actors who embrace liberal democracy rhetorically relate structures and citizenship, and for raising relevant questions for those who oppose it.

Educational sociology studies based on world culture theory emphasise commonality. They argue that structural reform has been driven, in both rhetoric and practice, by the growing dominance of liberal citizenship norms emphasising individual egalitarianism and education as a human right. This dominance means that liberal norms implicitly shape actors’ thinking, but also that actors must explicitly refer to them to gain legitimacy before national and international audiences that accept them (Furuta 2020; Jakobi, 2011). This framework can explain why parties across the aisle link their structural preferences to liberal citizenship norms, while avoiding mention of potentially discrediting illiberal norms.

Work rooted in the politics of education foregrounds competition. This work argues that political actors have incentives not only to follow global logics but also, if they have electoral ambitions in a democracy, to support policies and rhetorical framings that appeal to their core constituencies (Ansell, 2010; Hibbs, 1992; Gingrich and Giudici, 2025). These incentives can explain differences in party rhetoric. In Western democracies, left-wing parties have traditionally represented less affluent constituencies and public sector workers who benefit from less differentiated structures that minimise social inequality, and for whom calls for solidarity and unity are more appealing. Conversely, centre-right parties tend to cater to wealthier voters and business interests. These groups typically have privileged access to more prestigious categories of schooling in stratified and de-standardised systems. They are therefore more likely to be able to exercise rights and opportunities autonomously.

Taken together, approaches from educational sociology and politics provide a useful framework for theorising both similarities and differences in the ways in which the parties that have dominated Western European (education) politics since 1945 rhetorically mobilise citizenship norms to legitimise their position on educational structures. However, both approaches are based on assumptions that are specific to these political actors, that is, to parties that rhetorically embrace the precepts of liberal democracy and who compete in elections for the sake of victory. To what extent do they apply to the far right?

## Educational structures, citizenship norms and the European far right

The electoral and cultural rise of the far right in recent decades has sparked a flurry of research, particularly in politics and history (Mudde, 2019). Educational analysis is still in its infancy, but the field is growing rapidly. At present, it consists largely of single-case studies of specific parties (Giudici et al., 2025; Neumann, 2022) or comparative analysis of recent manifestos of radical-right populist parties (Berg et al., 2023). The focus on populist parties overlooks that, as a result of its marginalised position in the first decades after 1945, the far right has developed into a particularly heterogeneous movement, both ideologically and organisationally (Castelli Gattinara and Pirro, 2024; Mudde, 2007; von Beyme, 1988). This internal variation can be exploited analytically and should be addressed to gain a more accurate understanding of the movement’s views and strategies.

Political scientists define the far right as an ideological movement that espouses authoritarianism, nativism, and either illiberalism or anti-democracy (Carter, 2018; Mudde, 2019). Authoritarianism and illiberalism refer to a belief in a strictly ordered society, with harsh punishments for transgressions and no regard for individual rights. In terms of citizenship norms, they imply a social order-based understanding of participation in which individuals participate according to their assigned roles in the social structure. Nativism holds that social categories such as nationality, but also gender or race, are organic and transcendent realities. It therefore implies a unitary, anti-pluralist identification with society (Dalton, 2008; Denters et al., 2007). Illiberal citizenship norms are at the heart of the movement’s ideology. However, the extent to which we might expect representatives to embrace them when discussing educational structures varies across the movement.

The first relevant source of variation is strategic-ideological. By 1945, fascism had been largely discredited in Europe. But its activists had not disappeared. A group of intellectuals, mainly based in France and Italy, set about adapting the ideology to the post-WWII legal and cultural context. This work involved replacing the most shunned and, in some places, illegal tenets of fascism – imperialism, militarism and biological racism – with principles such as an elitist social order and unitary nationalism (Bar-On 2007; Mammone, 2015).

Parties across Western Europe, starting with the Italian MSI in 1946, have since adopted this variant of far-right ideology, which is closer to inter-war fascisms. Openly or implicitly rejecting democracy, they often do not prioritise electoral competition and instead use their organisations to connect with activists and spread their message. Until recently, they have remained electorally uncompetitive niche parties (Mudde, 2019). These parties and associated intellectuals are sometimes referred to as the first generation (von Beyme, 1988), neo-fascist (Minkenberg, 2000), or extreme variant of the contemporary European far right (Mudde, 2019). We use the latter most common term here.

Not everyone was satisfied with the far right being an ideologically committed niche force. In the 1960s, a group of intellectuals led by the French *Groupement de Recherche et d'Études pour la Civilisation Européenne* (GRECE) embarked on a project to make far-right ideology more electorally palatable. The result of this work was a new variant of far-right ideology that replaced elitism and anti-democratic beliefs with authoritarian illiberalism and nativism (Bar-On, 2007; Carter, 2018; Mudde, 2007).

In the 1980s, a new generation of parties began to incorporate this variant of far-right ideology into their programmes (Mudde, 2007; von Beyme, 1988). These parties had no reservations about democratic elections as a means of gaining power. In order to appeal to a cross-class electorate, including lower-class voters (Halikiopoulou and Vlandas, 2020), they adopted populist anti-establishment views that divided society into people and elites, and emphasised that politics should reflect the will of the former (Canovan, 1999; Mudde, 2007). Following Mudde (2019), we refer to this group as the radical right.

The second relevant source of variation within the far right is organisational. Because of their marginalisation in electoral and parliamentary politics, far-right parties have sought allies outside the party landscape. This specificity contributed not only to the internationalisation of the post-1945 European far right, but also to its resemblance to a social movement rather than a traditional party family. For this movement, street and intellectual activism are as important political tools as institutional (party) politics, including in education (Bar-On, 2007; Castelli Gattinara and Pirro, 2024; Giudici, 2021; Mammone, 2015).

In this constellation, organised intellectuals have played a particularly important role. Not only do they prepare the ideological ground on which parties can build. As many post-1945 democracies have introduced stricter rules on party competition, intellectual collectives have also taken on the task of spreading ideology and bringing about cultural change – a strategy that the movement itself calls ‘meta-politics’ or ‘right-wing Gramscism’ (Minkenberg, 2000).

The combination of these two sources of variation results in four types of actors: extreme-right intellectual collectives, extreme-right parties, radical-right intellectual collectives, and radical-right parties. From a purely ideological perspective, if these four groups rely on citizenship norms to justify their position on educational structures, we should expect them to refer to the norms associated with illiberalism. They should therefore rhetorically frame structures as a means of promoting social-order based participation and a unitary identification with society (Dalton, 2008; Heater, 1990; Wallace Goodman, 2022). However, considering the sociological and political perspectives introduced earlier, our predictions are more nuanced.

World culture and politics of education approaches assume that actors want to win elections and appear legitimate in a liberal global order. These assumptions also apply, to some extent, to radical-right parties. While world culture theorists have observed an international rise in illiberal attitudes (Schofer et al., 2022), particularly in Europe, demonstrating governability may still imply a need to demonstrate commitment to entrenched liberal norms. Politically, the strategy of radical right parties has been to win over ever broader sections of the electorate, which involves adopting positions and rhetorical frameworks that appeal to voters across class lines (Gingrich and Giudici, 2025). They therefore have less incentive to rhetorically frame structures as a way of creating different ranks of citizens, putting individuals in their place, and denying them plural forms of identification. We should expect this group to be less likely to refer to social order-based and unitary citizenship norms.

The assumptions of world culture and politics of education should be less relevant to radical-right intellectuals and extreme-right parties. While the former engage with global norms to advocate broader cultural change, they do not participate in elections. Extreme-right parties participate in elections, but to spread their ideology rather than to win. We should expect these groups to refer more to illiberal citizenship norms. The same assumptions apply least to extreme-right intellectuals, whose organisation and strategy depend neither on electoral nor on global incentives.

Are these predictions correct? If such variation can be observed across the movement, how do different groups qualitatively use citizenship norms to justify their positions on structures? The following section outlines our empirical strategy for answering these questions.

## Empirical Approach

This study aims to examine whether and how the far right uses citizenship norms to justify its preferences towards educational structures, in order to shed light on the logics underlying the educational approaches of the movement’s different strategic and organisational currents. For both parties and intellectual organisations, our analysis is based on original sources documenting their official and collective communication. Since these two types of actors communicate through different channels, our analysis relies on two types of data collection and sources.

In line with the research on party politics, we draw on political manifestos to capture party communication. Manifestos are documents published by a party organisation to communicate its preferences and ideas to the electorate after a programmatic change or before an election. They therefore document a party’s official line in a condensed format that is comparable across time and place (Spoon and Klüver, 2014).

Our manifesto dataset includes all extreme and radical right-wing parties that have held at least one seat in a national (and, in federalised countries, regional) parliament in Western Europe since 1946. We identified relevant parties by combining two authoritative sources: Mudde’s (2007) anthology of European far-right parties, which includes information on the early post-WWII period, and the populist dataset compiled by Rooduijn et al. (2019), which starts in 1989. We used the same sources, in addition to the case-based literature (Appendix Section 4), to classify parties as either extreme or radical right. This procedure resulted in an underrepresentation of extreme-right parties, and we added some locally successful parties to the list for comparison.

Our collection includes the manifestos of all listed parties from 1946 onwards.^1^ Some of these manifestos are available online, notably in the collection compiled by the MARPOR project (Volkens et al., 2020). The remaining manifestos were collected from national, regional and party repositories, some digital and some physical (for a list, see Appendix Section 2).

As they do not participate in elections, intellectual collectives are not obliged to publish manifestos. We therefore rely on the views expressed in the reviews and books published by their official publishing houses to determine their positions. Gathering this data required archival work and travel, so we focus on a selected sample of organisations that the literature considers to be particularly influential in shaping and disseminating the extreme and radical-right message (Bar-On, 2007; Mammone, 2015). These include the *Défense de l'Occident* group around Maurice Bardèche in France and the Italian *Istituto Nazionale di Studi Politici*, *GNOMES* and *Centro Studi Ordine Nuovo* for the extreme right. The radical right is represented by what is considered its undisputed intellectual leader (Bar-On, 2007), the French *Groupement de Recherche et d'Études pour la Civilisation Européenne*, and its German and Italian counterparts (*Nuova Destra, Neue Rechte*). Table 1 lists the actors analysed, their countries and periods of activity, while a full list of the periods and sources analysed can be found in the Appendix Section 3.

**Table 1.** Far-right actors included in the analysis according to type: Name, country and period of activity.

**Extreme-right parties**
Movimento Sociale Italiano (IT 1946–1994); Suomen Maaseudun Puolue/Perussuomalaiset (FI 1959–); Schweizer Demokraten/Nationale Aktion (CH 1961–); Nationaldemokratische Partei Deutschlands (DE, 1964–); Sverigedemokraterna (SE 1988–); Laikós Syndesmos – Chrysi Avgi (EL 1993–); Forza Nuova; (IT 1997–) Fiamma Tricolore (IT 1999–); British National Party (UK 2005–19); Casapound (IT 2008–2019); Ethniko Laiko Metopo (CY 2011); Fratelli d’Italia (IT 2012)
**Extreme-right intellectuals**
Centro Studi Ordine Nuovo (IT 1956–1969); Défense de l’Occident (FR 1968–1986); Istituto Nazionale di Studi Politici (IT 1960s); Gnomes (IT 1970–1980s)
**Radical-right parties**
Schweizerische Volkspartei (CH 1936–); Freiheitliche Partei Österreichs (AT 1956–); Fremskridtspartiet (DK 1972–); Front National/Rassemblement National (FR 1972–); Vlaams Blok/Vlaams Belang (BE 1974–); Centrumpartij (NL 1980–1986); Die Republikaner (DE 1983–); Centrum Democraten (NL 1984–2002); Deutsche Volksunion (DE 1987–2010); Auto-Partei/Freiheits-Partei (CH 1991); Lega dei Ticinesi (CH 1991–); Lega Nord/Lega (IT 1991–); Ny Demokrati (SE 1991–1994); United Kingdom Independence Party/Reform UK (UK 1993–); Alleanza Nazionale (IT 1994–2009); Dansk Folkeparti (DK 1995); Laikós Orthódoxos Synagermós (EL 2000–); List Pim Fortuyn (NL 2003–2008); Bündnis Zukunft Österreich (AT 2005–); Mouvement Citoyen Genevois (CH 2005–); Partij voor de Vrijheid (NL 2006–); Parti Populaire (BE 2009–2019); Alternative für Deutschland (DE 2013–); Vox (ES 2013–); Nye Borgerlige (DK 2015–); Forum voor Democratie (NL 2016–); Chega! (PT 2019–); Juiste Antwoord 2021 (NL 2020–)
**Radical-right intellectuals**
Groupement de recherche et d'études pour la civilisation Européenne (FR 1969–); Nuova Destra (IT 1977–); Neue Rechte (DE 1986–)

To build our source-base, we systematically scanned these groups’ official publications to identify texts mentioning educational structures, and included articles and books that did so in our database. This means that while mentioning educational structures was a criterion for a text to be included in this category of data, this is not the case for manifestos. Our text units are therefore manifestos and, for intellectual collectives, articles and books. A full list of sources can be found in the Appendix.

To systematically compare preferences, we chose a deductive variant of content analysis and assigned statements in both sources to the same pre-defined set of codes (George, 2009; Smith, 2000). We developed the codes based on the framework developed in Section 1.1. Thus, our codes capture two types of statements: citizenship norms and preferences towards educational structures. To capture citizenship norms, we coded statements that express either one of three ideals of participation (autonomy, solidarity, social order) or one of two ideals of society (unitary or pluralistic).

Capturing preferences towards educational structures is more complex. Actual education systems vary widely in terms of their stratification and standardisation; even systems with long periods of comprehensive schooling may be more or less internally stratified. In their communication, actors typically refer to the system that their domestic audiences know and that they want to change. To ensure comparability, we therefore coded the direction in which they want to change their respective status quo. This means that we coded statements that express a preference for policies that either increase or decrease the stratification (e.g. tracking, grading) or the standardisation (e.g. regulation of the private sector, school autonomy) of a particular education system. We also coded the logic by which policies are intended to differentiate students: ability, ascriptive (gender or ethnicity), individual preference, or collective needs of society. Table 2 briefly defines the codes, while more detailed definitions and examples for each code can be found in Appendix Section 1.

**Table 2.** Codebook: Codes with brief definitions.

Dimension	Codes	Definition
**Citizenship norms**		
Role and behaviour	Social order	Citizenship as duty, participation according to role in social order and hierarchy
	Autonomy	Citizenship as active and independent participation, challenging authorities
	Solidarity	Citizenship as mutual support and equal participation
Identification with collective	Unitary	Citizenship requires cohesion and identification with unified collective
	Pluralist	Citizenship requires tolerance for individual diversity and group identities
**Structural differentiation preferences**		
Stratification	Higher/more	More differentiation by increasing the number or hierarchy of regulated types of education provision and certificates
	Lower/less	Less differentiation by decreasing the number and hierarchy of regulated types of education provision and certificates
De-standardisation	Higher/more	More differentiation by state interventions aimed at increasing the uniformity of teaching provision across a territory (regardless of level of state authority)
	Lower/less	Less differentiation by limiting state interventions aimed at increasing the uniformity of teaching provision across a territory
	Selective	Selective differentiation by increasing state interventions aimed at increasing the uniformity of teaching provision for minority education (e.g. specific ethnic groups)
Differentiation logic	Abilities	Differentiate structures based on students’ talents and abilities
	Ascriptive criteria	Differentiate structures based on ascriptive criteria such as gender and ethnicity
	Individual preferences	Differentiate structures based on students’, parents’ or providers’ individual preferences
	Collective needs	Differentiate structures based on collective needs of society

We coded sentences referring to formal education, from primary to tertiary. To ensure reliability in the application of the coding system (Smith, 2000), both authors coded the entire dataset. We also regularly compared our coding to discuss discrepancies and refine definitions until we had a validated set of coded sources.

We then analysed these codes both quantitatively and qualitatively. The quantitative analysis shows the number and proportion of documents that mention each category of citizenship norms and structural preferences for the four groups. It therefore provides information on whether and to what extent parties or intellectuals choose to use these concepts rhetorically. The core of the analysis follows the rules of qualitative content analysis in exploring how sources relate preferences for differentiation to citizenship norms (George, 2009). We present the findings below.

## Findings

The analysis shows that, despite their ideological affinity with globally discredited and electorally fraught citizenship norms, far-right actors do rely on such norms to justify their preferences towards educational structures. This section illustrates how both the extent to which these norms and preferences are mentioned and the framing of their relationship varies across the movement, starting with the extreme right.

### Extreme-right rhetoric: Standardised stratification for social order and belonging

As Table 3 shows, social order is the main participation norm mentioned by all groups, but it is particularly dominant in extreme-right texts. In extreme-right *intellectual* texts, this norm is typically framed in explicitly anti-democratic terms and combined with a unitary understanding of identity. Both French and Italian publications describe citizenship as a duty to be fulfilled in the awareness that ‘before the Fatherland every individual personality disappears’ (Salmi, 1961: 89). Authors rail against political systems that empower ‘the masses’ (Cimmino, 1960: 213) and rely on ‘the omnipotence of numbers’ (Bardèche, 1961: 38) rather than the leadership of a select few.

**Table 3.** Citizenship norms.

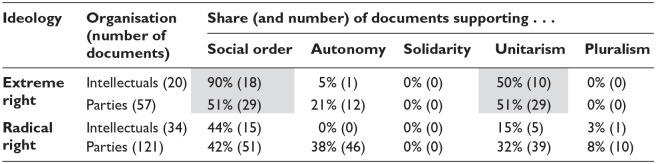

Shares above 50% are shown in grey.

Formal education has a key role to play in realising this understanding of citizenship. Extreme-right intellectual texts argue that the success of a society depends not on institutions of democratic governance, but on educational institutions designed to identify those who are fit to lead and to place them in positions of power, while giving everyone else ‘the feeling of being in their place in an accepted order’ (Bardèche, 1961: 190). They thus establish a direct link between citizenship norms and educational stratification. It is schooling which, ‘through its different streams and levels of study, decides who should go up and who should stay down’ (Travostini, 1970: 64). Accordingly, an overwhelming majority of these texts support increasing hierarchical stratification (85%, see Table 4). Tracking should begin early, in lower secondary or primary school, with the number, size and hierarchy of streams deriving directly from society’s need to fill different occupations and roles; ability and the collective are the main logics of selection endorsed by these texts, as shown in Table 5.

**Table 4.** Preferences for education structures.

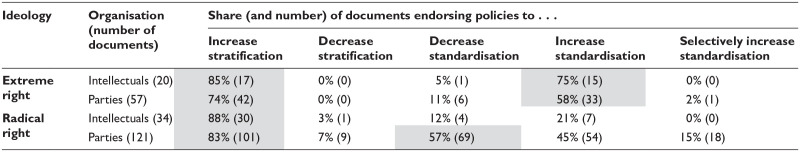

Shares above 50% are shown in grey.

**Table 5.** Preferences on logic of selection.

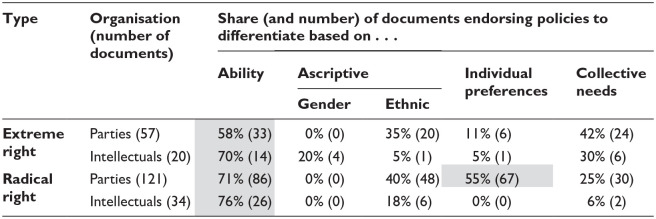

Shares above 50% are shown in grey.

The text of extreme-right intellectuals also relate standardisation directly to citizenship norms. They typically justify preferences for the state to enforce homogeneous standards with the need to ensure that citizens both identify with and participate in a unified national collective according to their assigned social roles. Indeed, these texts largely portray de-standardisation policies, such as increased school autonomy or private provision, as distortions of ability-based selection and integrated citizenry. More than that, they are a ‘moral slap in the face’ (Travostini, 1970: 81), potentially allowing someone to rely on their ‘bourgeois privilege’ (ibid.) to achieve a role that does not correspond to their innate talent. From this perspective, ‘selectivity is the fundamental concept of schooling’ (Bacci, 1971: 84), and compensatory programmes and rigorous selection must ensure that it is based only on aptitude, so that everyone fulfils the role in which they can best contribute to the collective.

Extreme-right parties express similar preferences on structures (Table 4). However, they justify standardised, stratified structures and social order in terms of what they describe as organic inequality rather than anti-democratic government. Here, early tracking and selection are needed to ‘recognise and respect the natural inequality of human beings’ (DE-NPD 1985). This is also the reason why structures must be designed in such a way as to create an elite and to instil in pupils a ‘sense of responsibility for their own country and people’ (CH-SD 2013). But the manifestos fall short of the openly anti-democratic rhetoric espoused by intellectuals. In an overlap with the radical right (see the next section), some more recent extreme-right manifestos even claim that schools need to build ‘critical capacity’ (IT-Fiamma 2008) and that ‘children should be taught how to think, rather than what to think’ (UK-BNP 2005). However, advocacy of such more autonomy-based understandings of citizenship does not fundamentally challenge this group’s preference for standardised stratification.

The caution of extreme-right parties in openly espousing anti-democratic views is reflected in their expressed preferences for standardisation. While most of their manifestos proclaim the need for the state to intervene and ensure standardised content and quality, they mention such intervention less frequently than intellectuals and do not openly oppose parental preferences. Some very recent manifestos even support school choice within a regulated system (11%, see Table 4, including IT-FDI 2022; UK-BNP 2005; IT-MSI 1994). By justifying such policies in terms of the need for parents to have the means to counter social mixing policies and ‘other integration experiments’ (SE-SD 2022), this turn provides evidence of de-standardisation acting as a substitute for (ethnic) stratification.

### Radical-right intellectuals: Stratified education for naturally unequal citizens

The reliance on supposedly natural inequality to justify structural preferences is even stronger in radical-right texts. Rather than legitimising stratification in terms of collective needs (Table 5), radical-right intellectuals tend to cite (selected) evidence from the sciences to justify the need for both social order and stratified education. GRECE often seeks support from evolutionary biologists, arguing that ‘elitism exists even in nature’ (De Benoist, 1978: 206). They also quote anthropologists to argue that ‘[t]he more advanced a society, the more hierarchical it becomes’ (Norey, 1974: 6).

Radical-right intellectuals apply this logic directly to education, arguing that systems that ‘refuse to select’ (Olles, 1998) are contrary to the needs of individuals and lead to mediocrity. Calls for meritocratic selection through gifted and talented programmes, early tracking, and the abolition of compensatory education are common in these texts. The only mention of a de-stratification policy concerns higher education, with the author claiming that future elites need a less specialised education to become capable leaders (Clemens, 1996).

The focus on natural inequality rather than collective governance and belonging means that radical-right intellectuals are more agnostic about standardisation. The issue is rarely mentioned and opinions are divided. Until the 2000s, most texts mentioning standardisation tend to support it. In 1984, GRECE stated its position as follows: ‘Differentiate public education: yes; privatise it: no’ (Valclérieux, 1984: 9). But some texts embrace private and autonomous education as a way of disrupting and re-stratifying the current education system – a position that becomes increasingly dominant in the 2000s. A 2002 GRECE text praised UK New Labour for allowing secondary schools to diversify their curriculum and work with private companies (Valclérieux, 2002). In doing so, intellectuals are paving the way for the much more de-standardisation-friendly views of radical right parties.

### Radical-right parties: Partisan politics and de-standardisation

The extreme-right parties and intellectuals, and radical-right intellectuals analysed so far differ mainly in rhetoric and justification, rather than in actual preferences. They all tend to associate the need for stratified and standardised provision (the latter is less pronounced in the texts of radical-right intellectuals) with citizenship norms based on social order and unitary belonging. Justifications shift from politics to natural inequality, but alternative citizenship norms and political preferences are rarely mentioned. Against this background, the manifestos of the radical right signal a new development.

In terms of citizenship norms, radical-right party manifestos also include social order-based understandings of participation (42%, see Table 3). Several parties rail against ‘egalitarianism’ (NL-CP 1982), arguing that ‘natural differences in talent are positive for the realisation of an ideal society in which one complements the other’ (ibid.). Others claim that elites are necessary to secure a country’s ‘economic and cultural position in the world’ (AT-FPOE 1999). These manifestos also advocate citizenship as ‘a moral attitude and a sense of duty towards the community’ (AT-FPOE 1957).

Like the other far-right groups, radical-right parties relate social order, inequality, and meritocracy directly to educational stratification. Qualitatively, however, their preferences are more nuanced and adapted to local contexts. Where stratification is more entrenched (e.g. Germany or Belgium), manifestos call for earlier and more hierarchical tracking, while in places with long-standing comprehensive reforms (e.g. the Nordic countries) parties emphasise internal differentiation and testing. Yet explicit support for de-stratification is rare (7%, Table 4).

What really distinguishes this group from the others is the high number of mentions of autonomy-based citizenship norms, which emphasise the creative role of new generations in shaping society (38%, Table 3). Many of these statements clearly adopt an oppositional logic, stating that schools must stop making students ‘accept whatever the government and teachers serve them’ (EL-LAOS 2007) or ‘forbid any critical attitude towards mass migration and multiculturalism’ (BE-VB 2019) and gender norms (DE-AfD 2016).

The texts relate this understanding to preferences for de-standardisation. These preferences are not monolithic; 57% of the radical-right manifestos analysed support policies that aim to de-standardise education, such as school choice and requiring parental consent for the teaching of certain topics (Table 4). At the same time, 45% also favour greater state control of provision through centralised testing, detailed curricula, monitoring and inspection to ‘ensure the continuity of the nation’ (FR-FN 1986). Many texts contain both preferences. As put by the Belgian Vlaams Belang, ‘We consider quality control of our education as a natural complement to private education’ (BE-VB 2007). Similarly, the French Front National in the 1980s placed vouchers, private provision, and parental choice at the heart of its platforms. It justified this position on the grounds of autonomy and the need to free parents from the allegedly left-wing control of state administrators and teachers’ unions, which ‘bear the seeds of totalitarianism’ (FR-FN 1986). As it has come closer to power, the party has become more pro-standardisation (Ferhat, 2025).

Qualitative analysis of these statements confirms the prediction that while de-standardisation is rhetorically linked to autonomous participation, it also acts as a complement to stratification in ensuring social order and unitary belonging to the native culture. This complementary function is first illustrated by the types of schooling that these parties want to protect from state-led standardisation, namely those closest to their own ideology. In places with strong religious (Catholic or Protestant) private schooling, such as the Low and Mediterranean countries, it is the character of these sectors that radical-right parties want to preserve: ‘Christian education is an indispensable cornerstone of the Dutch educational landscape’ (NL-FVD 2021). Elsewhere, the parties pin their hopes on community- and parent-run schools to counter ‘political pressure’ (DK-NB 2022) and ‘state propaganda’ (IT-LN 2022).

The second piece of evidence for the complementary function of de-standardisation is the radical right’s advocacy of selective de-standardisation policies, that is, policies aimed at specifically closing, regulating or defunding minority schools. Radical-right parties are the only group to mention such policies, and only recently, with the first instance of a selective standardisation policy mentioned in 2001. All these mentions target schools that are perceived as not conforming to the unitary identity of a community. In almost all cases, they are more or less veiled attacks on Muslim-majority schools, such as: ‘Freedom of education is a great thing. But there is no place for Islamic education’ (NL-PVV 2021).

The preferred logic of selection further underlines the use of de-standardisation as a means of stratifying society. As noted above, all other groups overwhelmingly favour selection based on ability and collective need (see, Table 5). For extreme-right intellectuals, this reasoning also underlies gender stratification. Girls and boys must be educated differently because they have different roles to play to best serve the collective, which ‘requires qualification from a young man but not from a girl’ (Travostini, 1970: 55). The radical right, on the other hand, focuses overwhelmingly on ethnic and linguistic criteria. Their argument is not based on ability – as when extreme-right intellectuals oppose ‘the cultural promotion of hitherto inferior people and races’ (Oxilia 1961: 326) – but on nativist claims that ethnic groups have different learning needs. De-standardisation of education would therefore be ‘in the academic interest of all ethnic groups’ (AT-FPOE 1990).

## Discussion and conclusion

This project sought to answer two questions. First, whether the post-1945 Western European far right, like other political actors, relates its preferences for educational structures citizenship norms. Second, whether the ways in which they do so can be understood through the lens of existing sociological and political theories of education policy. The analysis answers both questions in the affirmative, albeit with some limitations.

We find that the far-right actors analysed rhetorically relate their preferences towards educational structures to citizenship norms. Specifically, all four groups refer most frequently to the norms associated with illiberalism, espousing social order-based and almost exclusively unitary citizenship norms. However, in line with the expectations derived from the existing literature, both the extent to which these norms are mentioned in relation to structures, and the way in which this relationship is framed, varies across the movement.

Quantitatively, the farther far-right actors are from the electoral and global logics that the existing literature uses to theorise political rhetoric, the more likely they are to refer (exclusively) to illiberal citizenship norms; 90% of extreme-right intellectual texts support citizenship based on social order, while only 42% of texts published by radical-right parties do the same. No extreme-right intellectual texts show support for the more globally accepted autonomy-based citizenship norm, while 38% of texts from radical-right parties do.

Qualitatively, extreme-right intellectuals, the group least influenced by global and electoral logics, are the most resolute and explicit in arguing that highly standardised hierarchical stratification is necessary to ensure an anti-democratic social order that stifles pluralism and gives some individuals more participation rights than others. They relate this argument to theories of (autocratic) government. Extreme-right parties and radical-right intellectuals express similar preferences. However, their argument focuses on organic inequalities, rather than governance, as the main reason for structuring education in a hierarchical way. While these groups argue that stratification is necessary to promote talent and leadership, parties in particular tend to downplay the fact that such a system also implies non-elite routes, no exit options for parents and non-leadership roles for some of their potential voters.

Radical-right parties, on the other hand, do not only adopt a different framing, but also different preferences. Without abandoning the traditional support for social order, unitarism, stratification, and standardisation, this group mixes it with autonomy-based citizenship norms and support for de-standardisation through private, local, and parental control. Thus, support for tighter control of the curriculum and early tracking is combined with support for school autonomy and parental involvement, in order both to align rhetoric with international norms and to reassure voters that they will still have a say in their children’s education; if they are members of the cultural majority. Indeed, these texts are the only ones to endorse selective standardisation and, in particular, a system in which the state controls the education of non-native children more than others.

This is where the theoretical framework adopted shows its limitations. Theories developed on the basis of insights from parties that adhere to democratic and international norms can help us to understand why far-right actors seeking to engage more with the wider electorate and the public discourse may be motivated to downplay anti-democratic citizenship norms (as in the case of extreme-right parties and radical-right intellectuals) or to mix them with liberal notions of citizenship and alternative preferences (as in the case of radical-right parties). However, they are less suited to understanding the particular ways in which they do this.

Why have radical-right intellectuals replaced theories of government as frames of reference for thinking about citizenship and structures with anthropological and psychological theories that foreground individual inequalities? Why have radical-right parties chosen to endorse certain de-standardisation policies (e.g. choice) but not others (e.g. curriculum deregulation), and how have they decided to focus rhetorically on the need to selectively standardise the education of cultural minorities? Answering these and other questions requires the development of theories based specifically on these parties and actors, their strategies, incentives, and ideology. Such efforts must recognise that for actors such as the far right, specific logics and incentives may exist for the elaboration of education-related positional rhetoric. These logics may relate to increasingly popular illiberal global ‘alternative cultural frameworks’ (Schofer et al., 2022: 510) or the pursuit of different electoral strategies based on their populism, nativism, and authoritarianism, and the electoral coalitions they seek to represent (Canovan, 1999; Castelli Gattinara and Pirro, 2024; Mudde, 2019).

Further qualitative and in-depth research may be needed to shed light on these dynamics. Adopting a broader comparative approach, this study does not allow us to identify how particular contexts and traditions have shaped actors’ specific preferences and rhetoric. More context-sensitive case studies can provide a finer-grained analysis of the mechanisms and rationales that shape how political movements approach education. Building on our analysis, these may consider both the specific ideological approach a particular actor adopts and, if the actor participates in democratic politics, their relationship with the public and the electorate. The exclusive focus on structures also means that we neglect other dimensions of education policy, including those that far-right parties have been shown to be particularly concerned about, such as the curriculum (Berg et al., 2023).

Existing theories of political actors’ educational views, rhetoric and politics are largely built on insights from centre-right and centre-left parties and progressive movements (Niesz et al., 2018; Giudici, 2021). This study represents a novel attempt to integrate the far right into this type of analysis. It shows that – when it comes to understanding citizenship and structures – existing analytical frameworks can be applied to the far-right end of the political spectrum, while also demonstrating the need to develop new approaches to further our understanding of the educational views and strategies of such political actors. This kind of theorisation will become even more important if, as has been seen in other policy areas (Abou-Chadi and Krause, 2020), further actors increasingly adopt views and rhetoric developed by the far right, a dynamic that would reshape the logics of education politics more broadly. European education research should be equipped to both identify and theorise such developments.

## Supplemental Material

sj-docx-1-eer-10.1177_14749041241308600 – Supplemental material for Shaping illiberal citizenries: Far-right justifications of educational structures Supplemental material, sj-docx-1-eer-10.1177_14749041241308600 for Shaping illiberal citizenries: Far-right justifications of educational structures by Anja Giudici and Anna Pultar in European Educational Research Journal

## Notes

1. This article refers to manifestos by specifying the country of origin, the name of the party and the year of publication. For example, FR-FN 1976 indicates the manifesto published by the French Front National in 1976.
