# Supplementary material for: Shaping illiberal citizenries: Far-right justifications of educational structures
Source: Eur Educ Res J. 2024 Dec 24;25(1):126–45. doi: 10.1177/14749041241308600 (PMC12758647; doi:10.1177/14749041241308600)
Supplement: sj-docx-1-eer-10.1177_14749041241308600 – Supplemental material for Shaping illiberal citizenries: Far-right justifications of educational structures [file sj-docx-1-eer-10.1177_14749041241308600.docx]

Online Appendix

**Shaping Illiberal Citizenries:**

**Far-right Justifications of Educational Structures**

### Anja Giudici (Cardiff University) and Anna Pultar (Heriot Watt University) December 2024

[Section 1 | Codebook 2](#_TOC_250006)

[Section 2 | Organisations and Sources: Parties 7](#_TOC_250005)

[Section 3 | Organisations and Sources: Intellectual Collectives 11](#_TOC_250004)

- 1. [Sampling: Intellectual Collectives 11](#_TOC_250003)
  2. [Sources: Intellectual Collectives 12](#_TOC_250002)

[Section 4 | Selection of Literature Used to Classify Parties 15](#_TOC_250001)

[Section 5 | Reference List 17](#_TOC_250000)

# Section 1 | Codebook

The paper Shaping Illiberal Citizenries aims to explore the relationship between citizenship norms and policy preferences on educational structures in documents published by leading Western European parties and intellectuals after the Second World War. To this end, we systematically analysed the selected test to identify texts including:

- - 1. Citizenship norms. Following Tannenwald (2006), we define beliefs as ideas that can be either normative (how something should be) or causal (how something works). More specifically, citizenship norms are ideas about how citizens should or can participate in society. We further distinguish two dimensions of citizenship norms, namely ideas about the role and behaviour of citizens, and ideas about the way in which citizens should identify with the community.
    2. Structural preferences. Following Tannenwald (2006), we define policy preferences as actionable statements, i.e. sentences that support or oppose concrete decisions or actions. More specifically, structural preferences are policy preferences regarding the degree and type of organisational differentiation by which education systems should distribute content, certificate and opportunities across the student population - which can be more or less. We follow Allmendinger (1989) in distinguishing between preferences towards stratification (degree of tracking and hierarchy between tracks) and de-standardisation (degree of heterogeneity in provision) as two types of organisational differentiation. We code both the degree and the logic of differentiation supported by actors.

Our unit of analysis is documents, and we consider texts relating to all types of formal education except further and pre-primary education. This is because of these two sectors' generally informal nature in much of the post-WWII period, which makes them difficult to compare across countries.

Table 1 and Table 2 define the categories used to code citizenship norms and structural preferences in detail.

Table 1 Definition of Codes: Citizenship Norms

| **Codes** | **Definition** | **Examples** |
| --- | --- | --- |
| **Citizens’ role and behaviour** | | |
| Social Order | Normative or causal ideas as citizenship being a duty; about good citizens having to participate responsibly and according to their role; about social order and hierarchies defining how people should behave in society. | “The people represents the set of citizens worthy of the name and hierarchically organised according to an aristocratic substratum inherent in any political form of government” (Travestini 1970, 14; references are listed in section 3.2). |
| Autonomy | Normative or causal ideas as citizenship being about individual participation in politics and society, critical thought and challenging authorities, creating opportunities to participate. | “The young people of today are the decision-makers of tomorrow. They should be enabled to participate actively, responsibly and according to their abilities in the further development of democracy within our pluralistic society” (AT-FPÖ 1983). |
| Solidarity | Normative or causal ideas as citizenship being about understanding and supporting each other, having the resources to participate more equally in society. | No example in the sources |
| **Citizens’ identification with society** | | |
| Unitarism | Normative or causal ideas as citizenship being about individual participation in politics and society, critical thought and challenging authority, creating for participation. | “Launching this project presupposes the voluntary reintegration of pedagogy into its community and historical context ... Conversely, the educational process is meaningless if it does not transmit to the individual the cultural heritage of the communities of which he or she belongs” (Lecrozet & Valclerieux 1975, 9). |
| Pluralist | Normative or causal notions of citizenship as tolerance, respect and acceptance of social diversity in terms of individual and group identities. | “The Vlaams Blok opposes the destruction of the rich specificity of education as it had traditionally grown in Flanders. This is now being abandoned and replaced by a sausage, a kind of shapeless, odourless and tasteless sandwich meat in which unitary schools, compulsorily mixed, compulsorily populated with Moroccans and Turks, compulsorily squeezed into one unitary type, compulsorily accommodated in mergers” (BE-VB 1995). |

Table 2 Definition of Codes: Structural Preferences

| **Codes** | **Definition** | **Examples** |
| --- | --- | --- |
| **Stratification** | | |
| High/more | Support for policies that aim to increase the separation between different regulated types of educational provision and certificates, and to increase the hierarchy between such types.  Relevant policies include adding tracks, increasing differences between tracks, grouping and levels of instruction, increasing/maintaining selectivity through testing and grading - regardless of the logic of selection. | “There are two ways of avoiding 'wasting grey matter': accelerated studies, which allow the most gifted to 'skip' classes or do two in a single year; and grouping, which consists of bringing gifted pupils together under the aegis of specialist teachers” (De Benoist 1978, 207).  “As experience shows and modern science has convincingly demonstrated, people are unequal in terms of their talents and performance. Those who, despite this knowledge, build a unitary school and university system bear the responsibility for all the consequences, ranging from child behavioural disorders to addictions and juvenile delinquency. Lack of breadth of knowledge and lack of elites are another feature of this wrong policy” (DE-NPD 1985). |
| Low/less | Support for policies that aim to integrate types of provision and qualifications or reduce the hierarchy between them.  Relevant strategies include merging courses, reducing differences between courses, grouping and levels of teaching, reducing selectivity and removing testing and grading. | “Higher education is at the top of the list of diverse educational paths. This pinnacle must not be reserved for a relatively few, it should be within reach of all - even only unilaterally - gifted people and actually be reached by as many of them as possible” (AT-FPÖ 1970).  “The BZÖ steps for the Expansion of a common school of the six to fifteen year olds or the "new middle school", as they are in Carinthia is already successfully tested. This form of school stands for more equality of opportunity through individual support for students, internal differentiation without performance groups and modern, reformed teaching methods, reformed teaching methods such as team teaching” (AT-BZÖ 2008) |
| **De-standardisation** | | |
| High/more | Support for policies that limit the extent to which state authorities can increase the uniformity of education provision across a territory (regardless of the level of state authority).  Relevant policies include measures to de- standardise student composition (e.g., by increasing choice), curriculum and quality (e.g., by reducing inspection, liberalising curriculum regulations, or allowing private provision), and | “Expansion of university autonomy and responsibility with clear demarcation from ministerial administrative competence” (AT-FPÖ 1983).  “Private education is thus caught in an irreversible downward spiral in which it loses its uniqueness, its quality characteristic and its competitive advantage more and more. The ban on schools programming their own fields of study is a |

|  | teaching conditions (e.g., abolishing school uniforms). | hardly edifying example of this” (DE-VB 1999). |
| --- | --- | --- |
| Lower/less | Support for policies that enable public authorities to increase the uniformity of educational provision across an area (regardless of the level of state authority).  Relevant policies include measures to standardise student composition (e.g., by defining catchment areas or regulating admissions), curriculum and quality (e.g., by increasing textbook regulation or monitoring), and teaching conditions (e.g., by introducing school uniforms). | “Authority in the school should be reaffirmed as 'power of the school': of the whole school, considered in the organicity of its fundamental components, as an intermediary body configured in the state system as a fertile field of education and culture” (Siena 1972, 21).  “Use of a government-imposed uniform standard for the assessment of pupils (deliberations) and for the imposition of re- examinations, holiday tasks and study contracts” (BE-VB 1987). |
| Selective | Support for policies that allow state authorities to selectively increase the uniformity of educational provision for specific territories, types of education, or groups (regardless of the level of state authority).  Relevant policies include measures that standardise for selected schools the composition of the student body (e.g., by defining admission criteria for religious schools), the curriculum and quality (e.g., by introducing stricter inspection regimes for ethnic schools), and teaching conditions (e.g., by selectively banning religious symbols in schools). | “Enhanced funding and security oversight of mosques, Koranic schools, Islamic home education and other Islamic initiatives” (BE-VB 2019).  “No subsidies for Muslim free schools. We will not subsidise Muslim colleges and schools. Islam suppresses the democratic values on which Danish society is based, and Muslim schools should therefore not receive public funding” (DK-NB 2019). |
| **Logic of differentiation** | | |
| Abilities | Support for policies that differentiate structures and select on the basis of pupils' talents and abilities. | “However, we must not forget that aptitudes, even hereditary ones, are only potential. If they are not given the opportunity to express themselves, they atrophy ... To express themselves properly, gifts require an appropriate favourable environment. Hence the need for identification, selection and constant, sustained support” (De Benoist 1978, 206).  “The rigid class compositions according to age groups, which do not take into account the aptitudes and talents of the pupils, must be supplemented by talent and performance groups” (AT-FPÖ 1970). |
| Individual preferences | Support policies that differentiate structures based on the individual preferences and beliefs of parents, pupils or providers. | “In order to guarantee a high degree of flexibility, every compulsory school leaver should therefore be given an education voucher and the promotion of gifted students should be promoted. By means of the education cheque, compulsory school leavers would have the opportunity to prioritise their apprenticeship training according to their own priorities” (AT-FPÖ 1990). |

|  |  | “All people should have the opportunity to be educated and active according to their inclinations. The state must provide the basis for this. The SVP demands - that everyone should have the opportunity to pursue an education of their own choice according to their talents and inclinations” (SVP 1995). |
| --- | --- | --- |
| Ascriptive | Support for policies that differentiate structures and select on the basis of ascriptive criteria such as gender or ethnicity. | “Explicit recognition that the social destination of women required specific educational institutions” (Bacci 1971, 72).  “Any disadvantage of Austrian pupils due to a high proportion of foreign-language classmates is to be prevented by setting up parallel classes in the main subjects and joint teaching in secondary subjects organised according to mother-tongue aspects” (AT-FPÖ 1990). |
| Collective needs | Support for policies that differentiate structures and select on the basis of collective economic or social needs. | “The PP intends to use proactive measures (quotas, entrance exams, financial incentives, etc.) to encourage students to take up courses with a future, rather than dead-end routes” (BE-PP 2014).  “The courses offered should be increasingly geared to the demands of the market.  Students educated past economic needs do not create value, but on the contrary inflate the social and welfare state” (CH-SVP 2007). |

# Section 2 | Organisations and Sources: Parties

The study considers two types of far-right organisations: parties and intellectual collectives. t is sometimes disputed (especially by the parties themselves, Mair & Mudde 1998) which parties can be considered representative of far-right party families. In order to select the parties for this study, we have therefore relied on two authoritative academic sources on the European far right. These are Mudde’s 2007 volume *Populist Radical Right Parties in Europe* and Rooduijn et al.’s 2019 *Populist.* The latter is an online inventory of populist parties active after 1989, which also indicates which parties scholars generally consider to have far-right ideology.

We restrict the selection of parties to Western European countries, as the literature has shown that Eastern European far-right parties may represent different beliefs and policies (Mudde 2007). In order to focus on parties with at least some influence, we further restrict the sample to parties that have been represented at the national (or sub-national, in federal countries) level at some point since 1946. This criterion leads to an under-representation of 'old' far-right parties, which we have corrected by adding selected unsuccessful parties. These are marked with an asterisk (*) in Table 3, which contains a list of the parties analysed, their categorisation as new and old far right, and the time frame of their activity and analysis.

Almost all Western European countries have had an active far-right party in the post-WWII period. There are three exceptions. Ireland and Norway have no documented far-right party to date.^1^ The other is Luxembourg, where the party considered to represent the far-right family, the 1987-1994 National- Bewegong, never managed to elect representatives at either the national or local level.

Our main source of documents is the Comparative Manifesto Project Database MARPOR (Volkens et al. 2020), which can be accessed online (https://visuals.manifesto-project.wzb.eu) and provides access to digitised manifestos (either pdf or text or both) for most parties that managed to elect at least one person to the national parliament.

These are, firstly, manifestos of smaller parties and regional parties in federal countries and, secondly, very recent manifestos that have not yet been processed by the team. For the most part, we were able to retrieve recent manifestos from party websites and national repositories. For smaller party manifestos, we used national digital or onsite repositories and archives, specialist literature, and the Polidoc database of regional manifestos (http://polidoc.net). Table 3 lists the sources of documents for each country.

^1^ The Norwegian Fremskrittpartiet is sometimes considered to be to the right of the conservative party, but in the literature we use it is not considered to be an extreme right-wing party from an ideological perspective.

This strategy allowed us to collect manifestos for all relevant parties and years. The only party for which we could not find a manifesto is the Belgian National Front (1985-..., with one seat in 2003 and 2007 and no mention of education in its current online platform).

Table 3 Analysed Parties in Alphabetical Order

| **Party Name** | **Country** | **Activity** | **Analysed Years** | **Type** | **Source** |
| --- | --- | --- | --- | --- | --- |
| Alleanza Nazionale, AN | IT | 1994-  2009 | 1996-2006 | New | MARPOR |
| Alternative für Deutschland, AfD | DE | 2013- | 2013-2017 | New | MARPOR  [www.afd.de](http://www.afd.de/) |
| Auto-Partei/Freiheits-Partei, FPS | CH | 1985- | 1991 | New | MARPOR |
| British National Party, BNP* | UK | 1982- | 2005-2019 | Old | general-election-2010.co.uk https://bnp.org.uk |
| Bündnis Zukunft Österreich, BZOE | AT | 2005- | 2008-2012 | New | MARPOR |
| Casapound, CP* | IT | 2008-19 | 2018 | Old | [www.casapounditalia.org](http://www.casapounditalia.org/) |
| Centrum Democraten, CD | NL | 1984-  2002 | 1987-1994 | New | MARPOR |
| Centrumpartij, CP | NL | 1980-  1986 | 1982 | New | MARPOR |
| Chega! | PT | 2019- | 2019-2022 | New | MARPOR |
| Dansk Folkeparti, DF | DK | 1995- | 1998-2019^2^ | New | MARPOR |
| Deutsche Volksunion, DVU | DE | 1987-  2010 | 1998-2003 | New | [http://polidoc.net](http://polidoc.net/)  German National Library, Frankfurt a.M. |
| Die Republikaner, Rep | DE | 1983- | 1992-2017 | New | [http://polidoc.net](http://polidoc.net/)  German National Library, Frankfurt a.M.  MARPOR |
| Ellinki Lisi, EL | EL | 2016- | 2019 | New | MARPOR |
| Ethniko Laiko Metopo, ELAM | CY | 2011- | 2016 | Old | MARPOR |

^2^ 2015-2019 nothing on education

| Fiamma Tricolore, FT* | IT | 1999- | 2008-2022 | Old | [www.fiammatricolore.org](http://www.fiammatricolore.org/) [http://pinorauti.org](http://pinorauti.org/) |
| --- | --- | --- | --- | --- | --- |
| Forum voor Democratie, FVD | NL | 2016- | 2017-2021 | New | MARPOR |
| Forza Nuova, FoN* | IT | 1997- | 2018 | Old | [www.forzanuova1997.it](http://www.forzanuova1997.it/) |
| Fratelli d’Italia, FdI | IT | 2012- | 2013-2019 | Old | MARPOR  [www.fratelli-italia.it](http://www.fratelli-italia.it/) |
| Freiheitliche Partei Österreichs, FPÖ | AT | 1956- | 1956-2021 | New | MARPOR |
| Fremskridtspartiet, FrP | DK | 1972- | 1973-1998^3^ | New | MARPOR |
| Front National / Rassemblement National, FN/RN | FR | 1972- | 1974-2022 | New | MARPOR  French National Library, Paris  Sciences Po Library, Paris |
| Juiste Antwoord 2021, JA21 | NL | 2020- | 2021 | New | MARPOR |
| Laikós Orthódoxos Synagermós, LAOS | EL | 2000- | 2007-2012 | New | MARPOR |
| Laikós Syndesmos - Chrysi Avgi, XA | EL | 1993- | 2012-2015 | Old | MARPOR |
| Lega dei Ticinesi, LdT^4^ | CH | 1991- | 2007-2019 | New | MARPOR  https://lega-dei-ticinesi.ch |
| Lega Nord / Lega, LN | IT | 1991- | 1994-2022 | New | MARPOR  https://legaonline.it  Italian National Library, Florence |
| List Pim Fortuyn, LPF | NL | 2003-  2008 | 2003 | New | MARPOR |
| Mouvement Citoyen Genevois, MCG | CH | 2005- | 2011-2023 | New | MARPOR  https://mcge.ch |
| Movimento Sociale Italiano, MSI | IT | 1946-  1994 | 1948-1994 | Old | MARPOR  Italian National Library, Florence |
| Nationaldemokratische Partei Deutschlands, NPD* | DE | 1964- | 1967-2021 | Old | [http://polidoc.net](http://polidoc.net/) |

^3^ Then becomes electorally insignificant and often supports others on the right

^4^ 2019 nothing on education.

|  |  |  |  |  | German National Library, Frankfurt a.M. |
| --- | --- | --- | --- | --- | --- |
| Ny Demokrati, NyD | SE | 1991-  1994 | 1991 | New | MARPOR |
| Nye Borgerlige, NB | IT | 2015- | 2015-2022 | New | MARPOR |
| Parti Populaire, PP | BE | 2009-  2019 | 2014 | New | MARPOR |
| Partij voor de Vrijheid, PVV | NL | 2006- | 2006-2021 | New | MARPOR  [www.pvv.nl](http://www.pvv.nl/) |
| Schweizer Demokraten/Nationale Aktion, SD | CH | 1961- | 1991-2022 | Old | MARPOR  www.schweizer- demokraten.ch |
| Schweizerische Volkspartei, SVP | CH | 1936- | 1971-2019 | New | MARPOR  [www.svp.ch](http://www.svp.ch/) |
| Suomen Maaseudun Puolue / Perussuomalaiset, PS | FI | 1959- | 1970-2019 | Old | MARPOR |
| Sverigedemokraterna, SD | SE | 1988- | 1989-2022 | Old | MARPOR  https://snd.gu.se |
| United Kingdom Independence Party, UKIP | UK | 1993- | 2001-2022 | New | MARPOR  [www.ukip.org](http://www.ukip.org/) |
| Vlaams Blok / Vlaams Belang, VB | BE | 1974- | 1981-2019 | New | MARPOR |
| Vox | ES | 2013- | 2019 | New | MARPOR |

# Section 3 | Organisations and Sources: Intellectual Collectives

While party manifestos are public and widely available by virtue of their function, this is not the case for documents published by intellectual groups. These are often intended to form the basis for discussion and debate within a movement and to inform party policy. They are therefore aimed at insiders rather than the wider (voting) public. As a result, these documents are more heterogeneous in nature and more difficult to find and compare - which meant that we had to limit our research to selected cases.

## Sampling: Intellectual Collectives

In order to select relevant far-right intellectual collectives, we followed the same strategy that we used to select relevant parties. That is, we first relied on authoritative literature to identify the most influential old and new far-right collectives. Our main scholarly sources were Bar-On’s 2007 and Mammone’s 2015 intellectual histories of the post-WWII Western European far right. Table 4 lists the collectives identified by these authors and whether they are considered representatives of the old or new far right.

In a second step, we searched for sources documenting these groups' views on education and citizenship. As with political parties, our aim was to analyse the literature that would be expected to represent the collectives' unified and official views - rather than the potentially outlying views of individuals. We did this by focusing on three types of sources: (1) reviews published by the collectives; (2) books and treaties published by the official publishing houses of these collectives or included in their official curricula and reading lists; (3) books and treaties published by movement leaders. While a few documents were available online, most had to be located in archives and libraries in the country of origin of these groups, i.e. Germany, France and Italy.

Finally, we analysed the publications to identify articles or books that discussed educational structures. Section 3.2 lists the publications (which serve as units of analysis) included in the analysis based on these criteria.

Table 4 Analysed Intellectual Collectives in Alphabetical Order

| **Collective Name Publications** | **Country** | **Activity** | **Analysed Years** | **Type** | **Source** |
| --- | --- | --- | --- | --- | --- |
| **Centro Studi Ordine Nuovo** |  |  |  |  |  |
| Education-related literature in official reading list *Bibliografia Generale Di Orientamento Tradizionale*:  Travostini, Luciano. 1970. *L’educazione Di Stato*. 2nd ed. Imola: Coop. Galeati. | IT | 1956-  1969 | 1956-1969 | Old | Italian National Library, Florence  Archivio Fondo Ugo Spirito, Rome |

| **Défense de l’Occident**  Official review and literature:  *Bardèche, Maurice. 1961. Qu’est-Ce Que Le Fascisme? Paris: Les sept couleurs.*  Reviews: *Voix de de l’Occident (1962); Occident Universitè (1965), Dèfense de l’occident (1968-1986*) | FR | 1968-  1986 | 1968-1986 | Old | French National Library, Paris |
| --- | --- | --- | --- | --- | --- |
| **GNOMES**  Publications issued by its publishing house:  Bacci, Silverio. 1971. *Dalla Legge Casati alla Carta della Scuola.* Roma: Gnomes.  Bacci, Silverio. 1980. *Una politica per la scuola.* Roma: Gnomes.  Siena, Primo. 1972. *Riforma organica della scuola italiana.* Roma: Gnomes.  Totaro, Giuseppe. 1982. *La comunità educativa in Italia.* Roma: Gnomes. | IT | 1970-  1980s | 1970-  1980s | Old | Italian National Library, Florence  Archivio Fondo Ugo Spirito, Rome |
| **Groupement de recherche et d'études pour la civilisation Européenne**  Official reviews:  *Élements* (considered 1973-76; 1979-85;  1995-2002; 2006-07)  *Études et recherche* (considered 1974-77; 1983-85)  *Nouvelle education* (considered 1976-82) | FR | 1969-… | 1969-2010 | New | French National Library, Paris  Sciences Po Library, Paris |
| **Istituto Nazionale di Studi Politici**  INSPE. 1960. *Problemi Della Scuola Italiana, Atti Del II Convegno Nazionale Dell’INSPE*. Rocca San Casciano: Cappelli editore. | IT | 1960s | 1960s | Old | Italian National Library, Florence |
| **Nuova Destra**  Official reviews:  *Elementi* (considered 1978-79)  *La voce della fogna* (considered 1978-83) | IT | 1977-  1980s | 1977-83 | New | Italian National Library, Florence |
| **Neue Rechte** Official review: *Junge Freiheit* | DE | 1986-… | 1986-2020 | New | German National Library, Frankfurt  a.M. & online |

## Sources: Intellectual Collectives

Ahlers, Janina. 2000. Die Macht der Gene. *Junge Freiheit*, 14(25). Online, [www.junge.freiheit.de](http://www.junge.freiheit.de/) Andergaszt, Max. 1996. Auf dem Weg zurück zur Eliteuni? *Junge Freiheit,* 11(12), 11.

Bacci, Silverio. 1971. *Dalla Legge Casati alla Carta della Scuola.* Roma: Gnomes. Bacci, Silverio. 1980. *Una politica per la scuola.* Roma: Gnomes.

Bardèche, Maurice. 1961. *Qu’est-Ce Que Le Fascisme?* Paris: Les sept couleurs.

C. L., 1975. Rémy Chauvin et les surdoués. *Elements* 12, 22.

Cimmino, Nicola. 1961. Necessità e possibilità di una riforma della scuola Italiana. INSPE (eds), *Problemi della scuola Italiana* (211-223). Firenze: Cappelli Editore.

Clemens, Björn. 1996. Bildungsstandort Deutschland. *Junge Freiheit*, 11(20), 13.

D.O. 1970. Dix-sept propositions de “Defénse de l’Occident”. *Défense de l’Occident* 18(88), 77-80.

De Bella, Paride. 1961. Scuola di stato e scuola private. INSPE (eds), *Problemi della scuola Italiana* (251- 254). Firenze: Cappelli Editore.

De Benoist, Marcel. 1978a. Les surdoués. In Marcel de Benoist, *Vu de droite* (205-208)*.* Paris: Copernic. De Benoist, Marcel. 1978b. L’université en proie aux bêtes. In Marcel de Benoist, *Vu de droite* (208-)*.* Paris:

Copernic

De Herte, Robert. 1975. Des exemples plutôt que des leçons. *Elements* 12, 1. GENE 1976. La scolarisation des enfants immigrés. *GENE-Info* 1(1), 15-16. GENE 1976. Réforme Haby: les avant-projects. *GENE-Info* 1(1), 4-8.

GENE 1976. Université: la réforme du 2ème cycle. *GENE-Info* 1(1), 10-14. GENE 1977. La guerre des deux écoles. *Nouvelle Éducation*, (2), 3-4.

GENE 1977. La réforme Haby (suite). *Nouvelle Éducation*, (2), 5-7. GENE 1977. Le projet éducatif de la FEN. *Nouvelle Éducation*, (2), 9-10.

Ghisellini, Vinicio. 1961. Riforma della scuola o riforma dello Stato e della società. INSPE (eds), *Problemi della scuola Italiana* (270-273). Firenze: Cappelli Editore.

Glück, Erich. 1997. Bildungspolitik: Felher und Halbheiten. *Junge Freiheit,* 12(40). Online, [www.junge.freiheit.de](http://www.junge.freiheit.de/)

Ihls, Alexander. 1989. Bildungspolitische Alternativen. *Junge Freiheit,* 4(2), 1-2.

INSPE. 1961. La mozione finale. INSPE (eds), *Problemi della scuola Italiana* (399-404). Firenze: Cappelli Editore.

Kraus, Josef. 1998. Schule ist konservativ. *Junge Freiheit,* 13(39). Online, https://jungefreiheit.de

Lecrozet, Joël, and Valclerieux, Fabrice. 1975. Principles d’un renouveau pédagogique. *Elements* 12, 2-12.

Manieri, Gino. 1961. Scuola di Stato e scuola non di Stato al servizio della Nazione. INSPE (eds), *Problemi della scuola Italiana* (224-228). Firenze: Cappelli Editore.

Molau, Andreas. 1991. Die Begabten werden vergessen. *Junge Freiheit,* 6(6), 8. Molau, Andreas. 1994. Was uns Bildung Wert ist. *Junge Freiheit,* 9(5), 9.

Nardi, Marcello. 1961. Esigenze culturali ed esigenze sociali nel Progetto di riforma della scuola media.

INSPE (eds), *Problemi della scuola Italiana* (243-248). Firenze: Cappelli Editore.

Nasti, Agostino. 1961. Intorno alla presunta giustificazione sociale di un’assurda riforma. INSPE (eds),

*Problemi della scuola Italiana* (224-228). Firenze: Cappelli Editore.

Norey, Michel. 1974. Bilan des travaux du SER. *Etudes et recherches pour la civilisation européenne,* 1, 3- 12.

Olles, Werner. 1998. Note “mangelhaft”. *Junge Freiheit,* 13(*4*6). Online, https://jungefreiheit.de

Oxilia, Adolfo. 1961. Necessità dell’inutile Latino. INSPE (eds), *Problemi della scuola Italiana* (323-335).

Firenze: Cappelli Editore.

Pagliaro, Antonio. 1961. La formazione culturale e civile dell’uomo nella scuola italiana. INSPE (eds),

*Problemi della scuola Italiana* (25-40). Firenze: Cappelli Editore.

Paratore, Ettore. 1961. Il posto del Latino nella realtà della scuola e nell’esigenza della nostra tradizione culturale. INSPE (eds), *Problemi della scuola Italiana* (309-322). Firenze: Cappelli Editore.

Rimet, Michel. 1969. L’université à la croisée des chemins. *Défense de l’Occident* 82(17), 10-22.

s.n. 1968. La réforme de l’enseignmet. *Défense de l’Occident* 16(76), 94-96.

s.n. 1976. Ré-enracinement culture? *Elements,* 14-15, 2.

s.n. 1976. Vers l’école Moyenne. *Elements,* 14-15, 2.

s.n., 1991. Gegen die Milieutheorie. *Junge Freiheit,* 6(3), 8.

Salmi, Roberto. 1961. La formazione del Cittadino nella scuola primaria. INSPE (eds), *Problemi della scuola Italiana* (89-91). Firenze: Cappelli Editore.

Siena, Primo. 1972. *Riforma organica della scuola italiana.* Roma: Gnomes.

Storck, Wilhelm. 1997. Zürich zur Leistung. *Junge Freiheit,* 12(47). Online, https://jungefreiheit.de Totaro, Giuseppe. 1982. *La comunità educativa in Italia.* Roma: Gnomes.

Travostini, Luciano. 1970. *L’educazione Di Stato*. 2nd ed. Imola: Coop. Galeati. V., J-.C 1975. Intelligence: l’inné et l’acquis. *Elements* 12, 10.

Valclérieux, Fabrice 1979. Editorial. *Nouvelle Éducation*, (9), 4-5.

Valclérieux, Fabrice 1980. Crises et remedes de l’education. *Nouvelle Éducation*, (11), 2-3. Valclérieux, Fabrice 1984. L’enseignement en question. *Elements,* 50, 5-9.

Valclérieux, Fabrice, 1975. Phantasme de la pédagomanie et réalités de la pédagogie. *Etudes et recherches pour la civilisation européenne,* 2, 3-20.

Valclérieux, Fabrice, 1985. Education: la fin de l’égalitarisme. *Elements*, 52, 9-11. Valclérieux, Fabrice, 2002. Et si l’on suivait le modèle anglais? *Elements,* 105, 58. Valclérieux, Fabrice. 1983. L’école des chomeurs. *Elements,* 44, 33-35.

Valla, Jean-Claude. 1975. Une reforme qui s’impose: L’école. *Elements* 12, 13-16.

# Section 4 | Selection of Literature Used to Classify Parties

Albertazzi, Daniele. 2006. ‘The Lega Dei Ticinesi: The Embodiment of Populism’. *Politics* 26 (2): 133– 39.

Altermatt, Urs, and Hanspeter Kriesi, eds. 1995. *Rechtsextremismus in der Schweiz*. Verlag Neue Zürcher Zeitung.

Art, David. 2012. *Inside the Radical Right*. Cambridge University Press.

Backes, Uwe. 2018. ‘The Radical Right in Germany, Austria, and Switzerland’. In *The Oxford Handbook of the Radical Right,* edited by Jens Rydgren, 452–77. Oxford University Press.

Betz, Hans-Georg. 1990. ‘Politics of Resentment: Right-Wing Radicalism in West Germany’.

*Comparative Politics* 23 (1): 45–60.

Betz, Hans-Georg, and Stefan Immerfall, eds. 1998. *The New Politics of the Right. Neo-Populist Parties and Movements in Established Democracies*. St. Martin’s Press.

Beyme, Klaus von. 1988. ‘Right-Wing Extremism in Post-War Europe’. *West European Politics* 11 (2): 1– 18.

Birenbaum, Guy. 1992. *Le Front National en Politique*. Balland.

Blee, Kathleen M, and Kimberly A Creasap. 2017. ‘Conservative and Right-Wing Movements’. In *The Populist Radical Right*, edited by Cas Mudde, 200–218. Routledge.

Brauner-Orthen, Alice. 2001. *Die Neue Rechte in Deutschland*. Leske + Budrich.

Castelli Gattinara, Pietro, and Andrea L P Pirro. 2024. *Movement Parties of the Far Right.* Oxford University Press.

Cole, Alexandra. 2005. ‘Old or New Right? The Ideological Positioning of Parties of the Far Right’.

*European Journal of Political Research* 44:203–30.

Copsey, Nigel. 2007. ‘Changing Course or Changing Clothes? Reflections on the Ideological Evolution of the British National Party 1999-2006’. *Patterns of Prejudice* 41 (1): 61–82.

Copsey, Nigel. 2007. ‘Changing Course or Changing Clothes? Reflections on the Ideological Evolution of the British National Party 1999-2006’. *Patterns of Prejudice* 41 (1): 61–82.

Crépon, Sylvain, ed. 2015. *Les Faux-Semblants Du Front National*. Sciences Po.

Eatwell, Roger, and Noël O’Sullivan, eds. 1992. *The Nature of the Right*. Pinter Publishers.

Ferhat, Ismaïl. 2023. ‘For Things to Remain the Same, Everything Must Change? Studying National Front’s and National Rally’s Platforms on Education’. *Journal of Contemporary European Studies*. https://doi.org/10.1080/14782804.2023.2269378.

Ferraresi, Franco, ed. 1984. *La Destra Radicale*. Feltrinelli.

Froio, Caterina, Pietro Castelli Gattinara, Giorgia Bulli, and Matteo Albanese. 2020. *CasaPound Italia: Contemporary Extreme-Right Politics*. Routledge.

Goodwin, Matthew J, and James Dennison. 2018. ‘The Radical Right in the United Kingdom’. In *The Oxford Handbook of the Radical Right*, edited by Jens Rydgren, 521–44. Oxford University Press.

Gress, Franz, Hans-Gerad Jaschke, and Klaus Schönekäs. 1990. *Neue Rechte und Rechtsextremismus in Europa*. Springer.

Griffin, Roger. 1996. ‘The “post-Fascism” of the Alleanza Nazionale: A Case Study in Ideological Morpholgy’. *Journal of Political Ideologies* 1 (2): 123–45.

Gruber, Oliver, and Philipp Schnell. 2023. ‘Sticking to the Core or Going beyond? The Austrian Freedom Party’s Educational Approach in a Longitudinal Perspective’. Jo*urnal of Contemporary European Studies*. https://doi.org/10.1080/14782804.2023.2221188.

Holsteyn, Joop J. M. van. 2018. ‘The Radical Right in Belgium and the Netherlands’. In *The Oxford*

*Handbook of the Radical Right*, edited by Jens Rydgren, 478–504. Oxford University Press.

Ignazi, Piero. 1998. *Il Polo Escluso. Profilo Storico del Movimento Sociale Italiano*. Il Mulino. Ignazi, Piero. 2005. ‘Legitimation and Evolution on the Italian Right Wing: Social and Ideological

Repositioning of Alleanza Nazionale and the Lega Nord’. *South European Society & Politics* 10

(2): 333–49.

Kitschelt, Herbert. 1995. *The Radical Right in Western Europe: A Comparative Analysis*. University of Michigan Press.

Mammone, Andrea, Emmanuel Godin, and Brian Jenkins, eds. 2012. *Mapping the Extreme Right in Contemporary Europe*. Routledge.

Mammone, Andrea, Emmanuel Godin, and Brian Jenkins, eds. 2013. *Varieties of Right-Wing Extremism in Europe.* Routledge.

Mammone, Andrea. 2015. *Transnational Neofascism in France and Italy*. Cambridge University Press.

Mattei, Paola, and Giorgia Bulli. 2023. ‘The Case of the Education-Identity Nexus of the Northern League in Italy, 1994-2018’. *Journal of Contemporary European Studies*. https://doi.org/10.1080/14782804.2023.2212598.

Mayer, Nonna. 2018. ‘The Radical Right in France’. In *The Oxford Handbook of the Radical Right*, edited by Jens Rydgren, 433–52. Oxford University Press.

Mazzoleni, Oscar, and Carlo Ruzza. 2018. ‘Combining Regionalism and Nationalism: The Lega in Italy and the Lega Dei Ticinesi in Switzerland’. *Comparative European Politics* 16 (6): 976–92.

Merkl, P H, and Leonard Weinberg, eds. 1997. *The Revival of Right-Wing Extremism in the Nineties*.

Frank Cass.

Minkenberg, Michael. 1992. ‘The New Right in Germany’. *European Journal of Political Research* 22 (1): 55–81.

Mudde, Cas. 2019. *The Far Right Today*. Polity Press.

Neumann, Eszter, and Paweł Rudnicki. 2023. ‘Populist Radical-Right Governments in Central-Eastern Europe and Education Policy-Making: A Comparison of Hungary and Poland’. *Journal of Contemporary European Studies*. https://doi.org/10.1080/14782804.2023.2211935.

Pelinka, Anton. 2017. *The Haider Phenomenon*. Routledge.

Rasmussen, Palle. 2023. ‘Right-Wing Populist Education Policy in a Social Democratic Welfare State Context’. Journal of Contemporary European Studies. https://doi.org/10.1080/14782804.2023.2199144.

Ruzza, Carlo. 2018. ‘The Radical Right in Southern Europe’. In *The Oxford Handbook of the Radical Right*, edited by Jens Rydgren, 505–20. Oxford University Press.

Rydgren, Jens. 2010. ‘Radical Right-Wing Populism in Denmark and Sweden’. *SAIS Review* 30 (1): 57– 71.

Widfeldt, Anders. 2018. ‘The Radical Right in the Nordic Countries’. In *The Oxford Handbook of the Radical Right*, edited by Jens Rydgren, 545–64. Oxford University Press.

Zaslove, Andrej. 2009. ‘The Populist Radical Right: Ideology, Party Families and Core Principles’.

*Political Studies Review* 7 (3): 309–18.

# Section 5 | Reference List

Allmendinger, Jutta. 1989. “Educational Systems and Labor Market Outcomes.” *European Sociological Review* 5 (3): 231–50.

Bar-On, Tamir. 2007. *Where Have All the Fascists Gone?* London: Routledge.

Benoit, Kenneth, Thomas Bräuninger, and Marc Debus. 2009. “Challenges for estimating policy preferences: Announcing an open access archive of political documents.” *German Politics* 18 (3): 440-453.

Mair, Peter, and Cas Mudde. 1998. “The Party Family and Its Study.” *Annual Review of Political Science* 1 (1): 211–29.

Mammone, Andrea. 2015. *Transnational Neofascism in France and Italy*. Cambridge: Cambridge University Press.

Mudde, Cas. 2007. *Populist Radical Right Parties in Europe*. Cambridge: Cambridge University Press. Rooduijn, Matthijs, Stjin Van Kessel, Caterina Froio, Andrea Pirro, Sarah De Lange, Daphne Halikiopoulou,

Paul Lewis, Cas Mudde, and Paul Taggart. 2019. *The Populist*. [www.popu-list.org.](http://www.popu-list.org/)

Tannenwald, Nina. 2005. “Ideas and Explanation: Advancing the Theoretical Agenda.” *Journal of Cold War Studies* 7 (2): 13–42.

Volkens, Andrea, Tobias Burst, Werner Krause, Paola Lehmann, Theres Matthieß, Nicolas Merz, Sven Regel, Bernhard Weßels, and Lisa Zehnter. 2020. *The Manifesto Data Collection. Manifesto Project (MRG / CMP / MARPOR). Version 2020a*. Berlin: Wissenschaftszentrum Berlin für Sozialforschung (WZB).
